# COSMIC: exploring the world's knowledge of somatic mutations in human cancer

**DOI:** 10.1093/nar/gku1075

**Published:** 2014-10-29

**Authors:** Simon A. Forbes, David Beare, Prasad Gunasekaran, Kenric Leung, Nidhi Bindal, Harry Boutselakis, Minjie Ding, Sally Bamford, Charlotte Cole, Sari Ward, Chai Yin Kok, Mingming Jia, Tisham De, Jon W. Teague, Michael R. Stratton, Ultan McDermott, Peter J. Campbell

**Affiliations:** Cancer Genome Project, Wellcome Trust Sanger Institute, Wellcome Trust Genome Campus, Hinxton, Cambridge, UK, CB10 1SA.

## Abstract

COSMIC, the Catalogue Of Somatic Mutations In Cancer (http://cancer.sanger.ac.uk) is the world's largest and most comprehensive resource for exploring the impact of somatic mutations in human cancer. Our latest release (v70; Aug 2014) describes 2 002 811 coding point mutations in over one million tumor samples and across most human genes. To emphasize depth of knowledge on known cancer genes, mutation information is curated manually from the scientific literature, allowing very precise definitions of disease types and patient details. Combination of almost 20 000 published studies gives substantial resolution of how mutations and phenotypes relate in human cancer, providing insights into the stratification of mutations and biomarkers across cancer patient populations. Conversely, our curation of cancer genomes (over 12 000) emphasizes knowledge breadth, driving discovery of unrecognized cancer-driving hotspots and molecular targets. Our high-resolution curation approach is globally unique, giving substantial insight into molecular biomarkers in human oncology. In addition, COSMIC also details more than six million noncoding mutations, 10 534 gene fusions, 61 299 genome rearrangements, 695 504 abnormal copy number segments and 60 119 787 abnormal expression variants. All these types of somatic mutation are annotated to both the human genome and each affected coding gene, then correlated across disease and mutation types.

## INTRODUCTION

COSMIC is a database system designed to bring together the world's information on somatic mutations in human cancer into one single system and make it easily explorable. Gene-focused manual curation delivers deep mutation profiles on known cancer genes selected from the Cancer Gene Census (1) (http://cancer.sanger.ac.uk/cancergenome/projects/census/). These profiles, across more than 2500 human cancer diseases, allow deep stratification of which mutations are causing which cancers. To complement this knowledge depth, systematic curation of cancer genomes, both via publication and consortium data portals, generates huge breadth of knowledge across all somatic human genome annotations, providing substantial power to discover new cancer-causing events.

Since COSMIC launched in 2004 (2) detailing four cancer genes, the last 10 years have seen an enormous growth in cancer genetics and genomics, allowing COSMIC to now represent full literature curations of 136 genes and 12 542 cancer genomes (total numbers of data are shown in Table 1). Originally designed to detail simple coding gene point mutations, COSMIC now describes millions of coding mutations, noncoding mutations, genomic rearrangements, fusion genes, copy number abnormalities and gene expression variants across the human genome.

**Table 1.** Total contents in version 70 of the COSMIC database, the August 2014 release

Genes (transcripts)	28 735
Tumor samples	1 029 547
Coding mutations	2 002 811
Curated publications	19 703
Fusion mutations	10 435
Genomic rearrangements	61 299
Whole genomes	12 542
Copy number aberrations	695 504
Gene expression variants	60 119 787

## DATABASE CONTENT

Curation of published cancer mutation data is achieved via two complementary approaches. In order to obtain great depth of knowledge on key cancer genes, all appropriate literature is identified for each gene, then subjected to manual curation. This manual approach allows the capture of very high detail across mutation positions, disease descriptions and other patient and population data (such as age, ethnicity and therapeutic regime). Over 2500 cancer disease classifications are currently described in COSMIC, from 47 primary tissue types, and manual curation is the only way to capture the level of detail required to define these populations. Manual curation additionally provides improved quality control over systematic approaches. While gene, nucleotide and vocabulary details can be checked automatically, experienced curators are much better at identifying inconsistencies or errors in publications, allowing the rejection of untrustworthy, incomplete or unspecific data sources; over 30% of the 25 715 papers so far scrutinized by COSMIC have been rejected. New genes are included in COSMIC only when curation of their literature is exhausted, and the mutation patterns are as up-to-date as possible. After initial release, information for these genes will be updated as new papers are published.

Complementary to the manual curation effort, a semi-automated approach has been developed for curation of large cancer genome (and exome) data sets. Data sources are identified from the published literature and online data portals. Over 300 cancer genome publications have now been curated, and COSMIC includes substantial data sets from The Cancer Genome Atlas (3) (TCGA; http://cancergenome.nih.gov) and International Cancer Genome Consortium (4) (ICGC; https://dcc.icgc.org) projects. Approximately half of COSMIC's cancer genomes are curated from these consortium data portals, the other half from curations of published literature. The details of samples and disease descriptions are curated into COSMIC manually, and the mutations, usually supplied as genomic co-ordinates, are annotated automatically via a software pipeline using Ensembl genome annotations (5) (http://www.ensembl.org). This utilizes custom software similar to the Variant Effect Predictor (VEP; 6) to identify the positions of coding mutations as well as consequence annotations. Somatic mutations in cancer are now described across almost all human genes.

While genome-wide resequencing is becoming a standard technology in cancer genetics, the methodologies are still imperfect, although rapidly improving. In these experiments, sequencing coverage is rarely complete, with GC-rich regions particularly suffering dropout (7). It is therefore often difficult to identify every genomic variant, or determine whether a sample is wild-type or simply not assessed at any given position. In the absence of sample-specific coverage information (or raw data to re-evaluate), COSMIC makes the standard assumption that every gene has been evaluated in every sample, and calculates mutation rates accordingly. All curated data in COSMIC is referenced, allowing investigators to independently verify their findings in greater detail. However, ‘*COSMIC cell line project*’ contains pre-publication exome resequencing results; to support research across these resources, raw data are being prepared for public release.

During its 10-year existence, the main focus of COSMIC has been the aggregation of point mutation data across genes and genomes. In addition to this, manual curation efforts include the description of fusion genes. Often observed in cancer, these mutations result from genomic rearrangements which usually translocate two coding domains close to each other so that they form a single mutant transcript driving tumourigenesis. Current curations focus on solid tumor fusions, with an intent to begin curating blood cancer fusions when the majority of solid tumor mutations are represented in COSMIC. All manual curation is driven by the Cancer Gene Census, a list of genes (currently 522) with substantial literature describing their impact in cancer development, which diseases are caused, and indications of the mechanism involved.

As the genomic approach to cancer genetics matures, a number of complimentary genome-wide annotations are adding substantial context to the understanding of mutation burden, and we are expanding COSMIC to accommodate these. Copy number alterations are well documented in cancer, with genomic amplifications and deletions regularly driving oncogenesis. Currently, the two cancer genome consortia are releasing substantial copy number (CN) information in regularly formatted data sets, and we have incorporated this into COSMIC. Gene expression variants are also regularly used to identify oncogenic drivers, with significantly increased or decreased levels of expression across sample cohorts identifying a driver signature. Again, cancer genome consortia have regularized their data output into standard formats, enabling our regular interpretation of these data into each COSMIC release. Extensive annotations across all the described data types are available in the current release (v70; August 2014), and will be updated with additional information in future releases.

## DATA ACCESS

The data in COSMIC are available in a number of different ways. Most accessibly, a custom website is available (http://cancer.sanger.ac.uk) which displays the information in a number of graphic and tabulated views, making it easily explorable. The data are also available via a BioMart, (8) for programmatic access or downloads of user-specified data subsets. The entire COSMIC database is also available, after registration, for download in several forms including CSV and VCF formatted datasheets, or a full export of the entire Oracle database.

### Website

**Figure 1.**
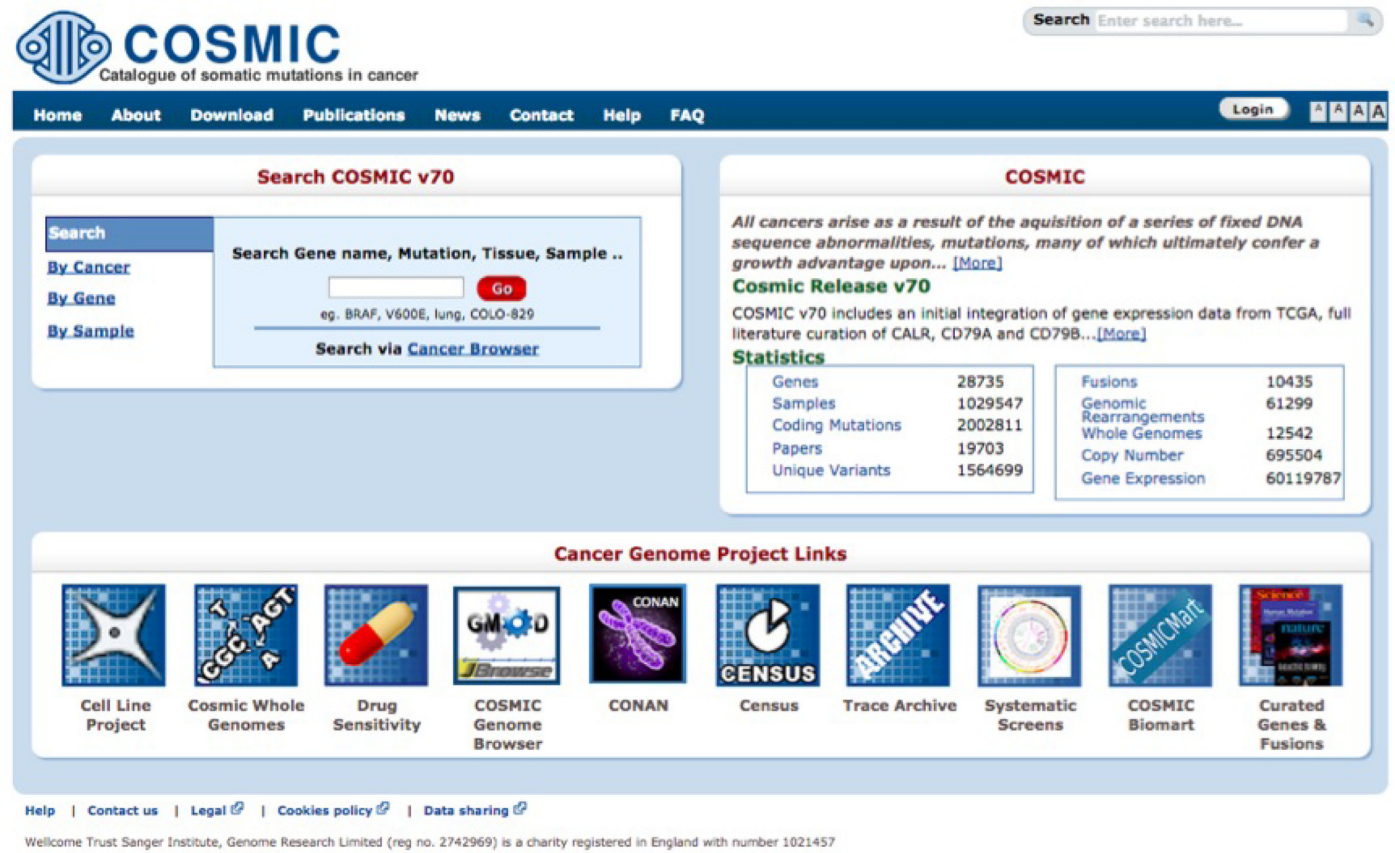
COSMIC website front page. Search options are presented in the left hand panel, descriptions of the content in the right side panel. The lower panel details related websites and other components of COSMIC. The dark bar at the top provides primary navigation to Help, Downloads and other descriptive content as well as a Contact link to the COSMIC helpdesk. Primary access to COSMIC is via the Search box in the left side panel, accepting multiple parameters including gene names, disease descriptions, mutation syntax and stable COSMIC IDs. ‘Search via Cancer Browser’ raises a new page providing navigation of mutation spectra behind thousands of cancer disease classifications.

The COSMIC website is available at http://cancer.sanger.ac.uk. Designed to make entry to COSMIC easy via one search box, the homepage (Figure 1) also provides access to a number of related resources. Three parallel websites allow the exploration of components of the COSMIC system. ‘*COSMIC cell line project*’ exclusively displays the results of genomic analysis across a large set of common cancer cell lines, currently numbering 1015 but expected to grow toward 1500. ‘*COSMIC whole genomes*’ displays only the genome-wide tumor analyses integrated into COSMIC, providing a view across the breadth of cancer genome data without any specific biases introduced via literature curation. *‘COSMIC*’ displays all the data brought into the system across the project's life, including cell lines, whole genomes and all genome-wide and gene-specific literature curations. Additionally, ‘*COSMIC genome browser*’ provides a genomic view across all COSMIC data types, aligned with many annotations from Ensembl, including noncoding RNAs (not yet included in the COSMIC website). ‘*Census*’ shows a listing of all the genes in the Cancer Gene Census including the details on disease causation and mutation mechanisms. Finally, ‘*Drug Sensitivity*’ links to a parallel resource in the team, describing the relationship, across more than 700 cell lines, between original disease, mutant genotype and response to a range of anticancer drugs. (9).

**Figure 2.**
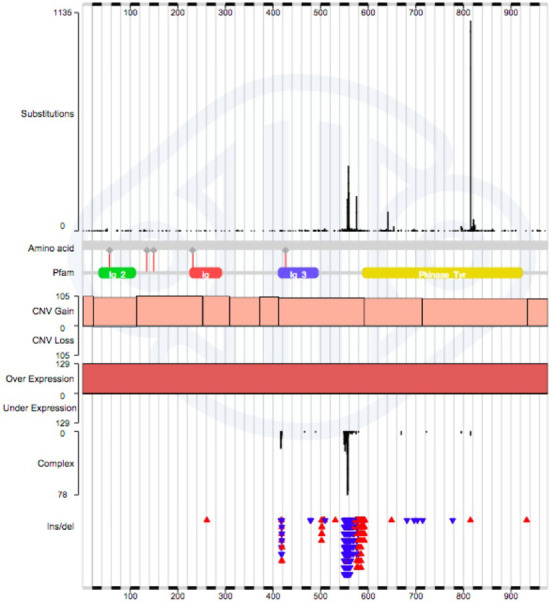
Full mutation distribution across all tissues and cancer diseases for the KIT gene. The X-axis describes the full length of the gene's coding sequence, and is zoomable (click & drag) to resolve amino acid or nucleotide sequences. In each section of data, the vertical height is kept static, while the scale changes according to the amount of data displayed. From the top down, the following mutation types are displayed: Single base substitutions, gene sequence, PFAM representation of peptide structure, copy number gain (pink)/loss (blue), gene over-(red)/under-(green) expression, multinucleotide substitutions (‘complex’), simple insertions (red triangles) and deletions (blue triangles).

**Figure 3.**
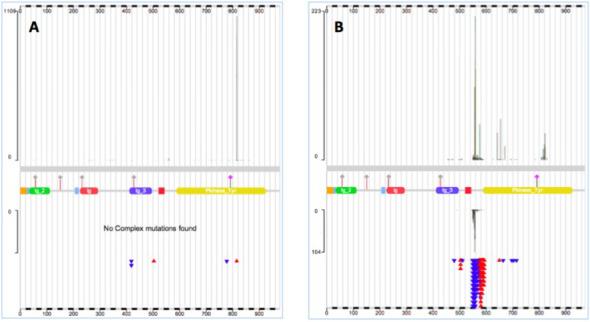
Example use of filters on the Gene Analysis page to explore disease-specific mutation burden, comparing the mutation profile of the KIT gene in two different tumor types, (**A**) Hematological and lymphoid: mast cell neoplasm and (**B**) Soft tissue: gastrointestinal stromal tumor. Substantial differences are very clearly shown in the mutation peaks between the two diseases in this gene, suggesting molecular biomarkers which may be exploited diagnostically, or in pharmaceutical target validation.

Navigation through the COSMIC website largely follows the selection of a single gene or disease, most easily typed in through the single Search box. The Search will respond with a list of items in COSMIC matching the input, offering matching gene names, mutations, samples, disease descriptions and even paper titles. Upon selection of any of these entities, a new page will appear showing an overview of its contents. If a gene was selected, the Gene Overview page will describe basic gene details together with results of drug sensitivity screens and a COSMIC genome browser describing all mutations around the gene's genomic footprint. Clicking on the ‘Histogram’ icon leads to a display of the mutation distribution across the gene, detailing the position and counts of point mutations, deletions, insertion copy number aberrations and expression abnormalities (Figure 2). This is the key graphic for exploring gene-specific mutation data, and a number of selections are available to filter the data, including tissue/disease type, tissue source, mutation type, somatic status and gene co-ordinate. The graphic itself is zoomable, by clicking and dragging the mouse cursor across the region of interest. Other tabs on this page describe further information (such as fusion mutations involving the gene selected), or offer further ways to explore the gene's data. Often the most useful of these is ‘Tissue’, which details tissue-specific mutation frequencies with counts and mini-histograms. Clicking on a count or a histogram block shows a tabulation of all the data behind that choice; clicking on a tissue name recalculates the table showing a much more detailed breakdown of diseases under that tissue, allowing much deeper exploration of mutation trends in that population. The Histogram and Tissue tabs are linked, with selections in one tab affecting the display of the other, such that the selection of ‘Soft Tissue: GastroIntestinal Stromal Tumor’ (GIST) in the tissue tab will recalculate the histogram to only show the mutation profile of that disease. Comparing the mutation distribution in *KIT* for GIST and ‘Haem: Mast Cell Neoplasm’ shows how useful such graphical exploration can be in exploring the mutation burden across cancer populations to identify novel targets and biomarkers (Figure 3).

**Figure 4.**
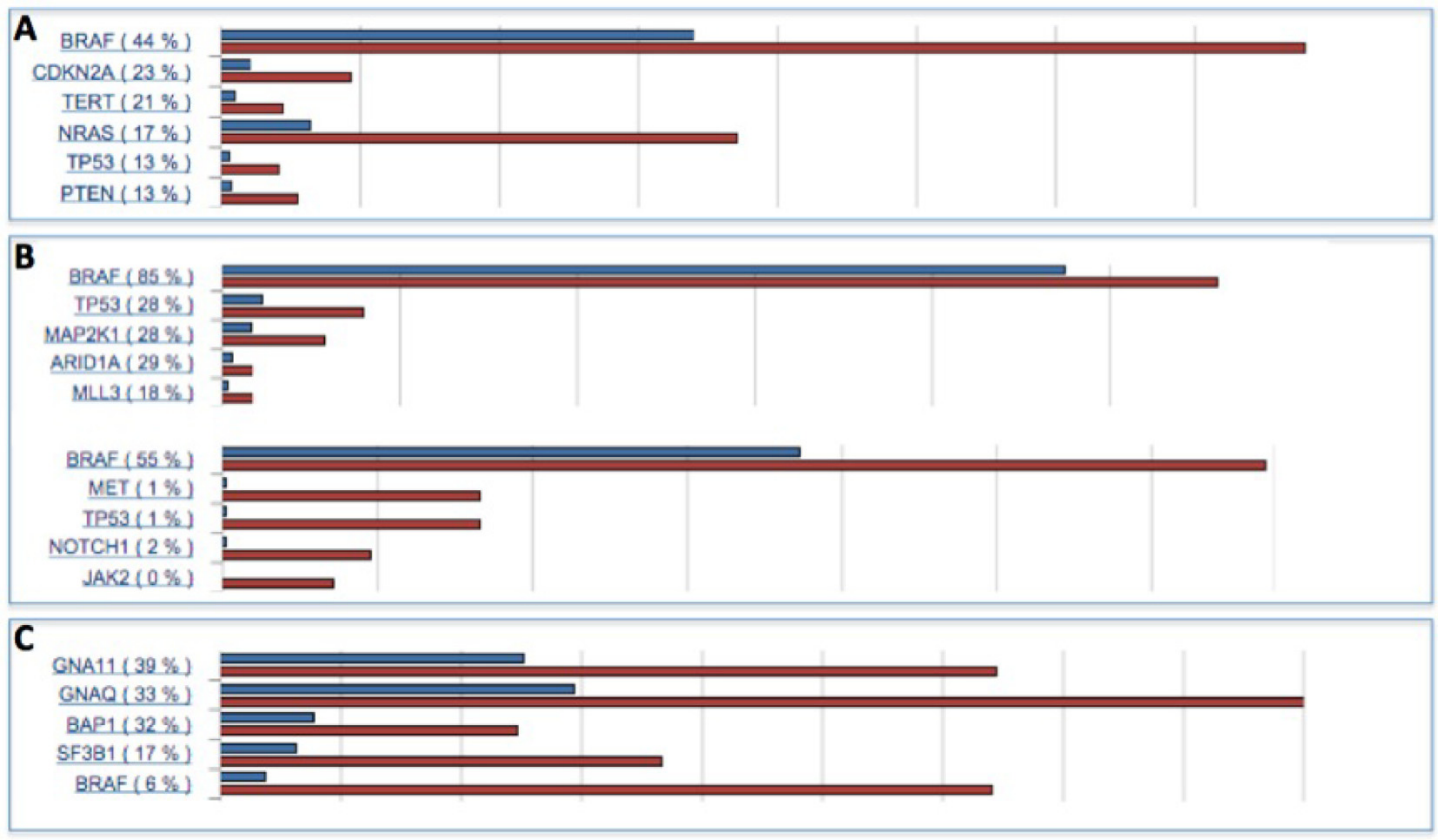
Histograms from the Cancer Browser, describing the most mutated top few genes on (**A**) skin melanoma (*n* = 9136), (**B**) rare blood cancers hairy cell leukemia (*n* = 514); Langerhans cell histiocytosis (*n* = 188) and (C) uveal melanoma (*n* = 714). Red bars represent the number of samples tested for each gene in the selected disease (‘*n*’), while blue bars represent the number of samples mutated; mutation rates simply calculate n_mutated/n_tested in each case. In this example, BRAF is a well known driver of skin melanoma, mutated in 44% of tumors tested (A). However, BRAF mutations are found at a much higher rate in very restricted populations with rare blood cancers (B). The low mutation frequency of BRAF in uveal (Eye) melanoma (6%) suggests very different genetic mechanisms behind this disease.

If COSMIC is being navigated by disease, either the Search box or the ‘Cancer Browser’ (http://cancer.sanger.ac.uk/cosmic/browse/tissue) will provide helpful access to over 2500 cancer disease classifications. After selection of a disease, pressing ‘Go’ will reveal multiple visualizations of the mutation patterns behind the disease of choice. Initially a simple histogram will display the top few (usually 20) highest mutated genes found in the disease (Figure 4), with links into the gene histogram page, to explore each gene in more detail. ‘Mutation Matrix’ will display a representation of the 200 most mutated samples and 20 most mutated genes for this disease, showing all mutation types in one image. Other graphics and tabulations provide additional ways to explore or detail the mutation information behind each disease.

Throughout the COSMIC website, graphic presentations are accompanied by data tabulations to enable complete access to the underlying data. This gives the graphics and interpretations on the website real transparency, since it allows the independent evaluation of each presentation. The display of data source references (largely Pubmed IDs) additionally allows independent evaluation of each publication's curation. Each tabulation is presented in the same format, with sortable columns and exportable contents. Clicking on a column title will sort the entire table on that column in ascending order, clicking on the title again re-sorts in descending order. This also works for the histogram blocks in the Tissue tab on the Gene Analysis page. Each table is also searchable, with a text box in the top right-hand corner available to enter search terms, to which the table will dynamically respond. Links are also available to download the entire contents of each table in Excel-compatible CSV format.

While the COSMIC website focuses on a genic presentation of cancer mutation data, cancer genomes are describing many more noncoding mutations and structural breakpoints than coding events. In order to make this information explorable in a genomic perspective, a COSMIC genome browser is now available. Initially conceived to add a genomic context to the Gene pages in the COSMIC website, it can be used independently, to explore all data types in COSMIC by typing a gene name or genomic coordinate in the search bar at the top of the page (http://cancer.sanger.ac.uk/jbrowse?data=data/json/cosmic&loc=3:10183532..10191649). Not all information is shown by default, as the page can become very cluttered, so it has been broken into ‘tracks’ which can be independently turned on and off. These are listed on the left hand side, with toggle buttons. Most significantly, this browser provides the ability to examine the coincidence of noncoding mutations or breakpoints with noncoding RNAs or other genomic annotations from the Ensembl database.

### Downloads

While the COSMIC website makes the entire database easily navigable with graphical presentations, it cannot provide easy methods to ask any complex bioinformatic question. For deeper mining of the information in COSMIC, the database is made available to download in multiple formats (http://cancer.sanger.ac.uk/cancergenome/projects/cosmic/download), for which registration is required. For fairly simple programmatic interpretation, all the information is available in preformatted CSV datasheets, some running to millions of rows. For point mutation data, VCF format is also available. For more complex data integrations, the entire database is available in its native Oracle format, either as ‘exp’ dump or ‘datapump’ formats. In addition to download files, a Biomart instance ‘COSMICMart’ (http://cancer.sanger.ac.uk/biomart/martview) is available which allows direct programmatic access alongside data filtering and mining tools.

## FUTURE WORK

**Figure 5.**
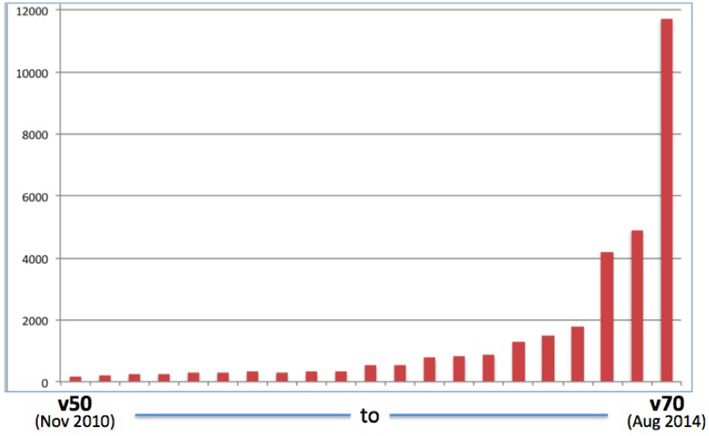
Growth of COSMIC database size (in Mb, of the Oracle ‘exp’ export file) between November 2010 and August 2014, emphasizing the rapid expansion as COSMIC reflects the data generated by cancer genome studies.

As genomic resequencing of large tumor cohorts matures, huge amounts of data are being rapidly produced, presenting many new opportunities to discover new oncogenic alleles across the human genome. Combining cancer genome annotations from several data types with extensive manual curations has driven a large growth of the COSMIC database across the last 4 years (Figure 5). The download size of the COSMIC database in November 2010 was 191 Mb (v50), but the v70 release (Aug 2014) had grown to 11.74Gb, requiring much more substantial download infrastructure as well as time. As the output of cancer genome publications and consortia expands, this growth trend is expected to continue.

Data curation is still the key ingredient in COSMIC, especially manual interpretation of the scientific literature; no other database is addressing the breadth of cancer genetics literature to the same extent as COSMIC. With a strong commitment to manual curation, this will be continued and expanded, focusing primarily on coverage of novel cancer genes. Genes already released in COSMIC will also be addressed, to ensure their mutation profiles are representative of up-to-date knowledge. Cancer genome curation will also be maintained, blending semi-automated interpretation of published data sets with the output of major cancer genome consortia. While current data sources include TCGA and ICGC, similar new data sources are regularly sought. Consortia project goals suggest future release of increasingly large data sets, and systems have been built to ensure COSMIC can handle this output. In between these two extremes, wide targeted gene screens, with several hundred genes tested through large tumor cohorts, are an emerging feature in cancer genetics, and this information will be curated through automated processes, as the data sets are often too large for effective manual curation.

In addition to showing point mutations on coding genes, the COSMIC genome browser allows the exploration of noncoding variants and their presence around noncoding genomic annotations, particularly noncoding RNAs. Automated curation systems will be adapted to define the effect of these mutations on ncRNAs, making these interpretations available in COSMIC. While additional data types such as copy number and gene expression are addressed in COSMIC, we will continue to find better ways of annotating variants, since interpretation of these data sets is a fast-changing field. DNA methylation data are also being output from cancer genome consortia, and ways of interpreting this are being explored to include this information in a future COSMIC release.

Cancer genomes are remarkably noisy sources of data, often producing hundreds or thousands of point mutations, copy number aberrations and gene expression variants per tumor, most of which have no effect on the development of disease. We are adapting our curation processes to reduce this noise and highlight high-value information. While all published information is included in the COSMIC database, SNPs and germline mutations are tagged, allowing them to be removed from the COSMIC website. Samples with over 20 000 point mutations, none of which have been validated are excluded from curation as their noise vastly outweighs their signal. Copy number annotations are split into numeric and descriptive data sets, the former with full details on absolute copy number at each locus, the latter simply annotating regions of ‘gain’ and ‘loss’. The numeric data sets are considered high value and are used by default to drive the copy number data on the website; the descriptive data are by default excluded, but an opt-in visualization toggle is offered. These noise reduction and quality control systems will be adapted and enhanced to maximize the utility of the data in COSMIC.

Identifying genes and mutations driving cancer is a goal of most COSMIC users, and several analytic methods are beginning to mature which might allow their systematic annotation, for instance the OncoDrive suite (10), MutSigCV (11), FATHMM (12). These algorithms are being scrutinized to see how they may be used to create a secondary annotation layer across the COSMIC database, significantly improving the identification of functional driver mutations in oncogenesis. COSMIC is the largest cancer genetics database in the world, and its growth and functional annotation will significantly enhance its support for cancer genetics research and druggable target identification as genetics increasingly grows to underpin clinical treatment.
